# Breast augmentation and sensory outcomes: A systematic review of surgical techniques and their impact on nipple-areola complex and breast-skin sensibility

**DOI:** 10.1016/j.jpra.2026.09.028

**Published:** 2026-09-29

**Authors:** Elie Ghadban, Cesar Ghadbane, Tigresse Boutros, Mohammad Ali Almokdad, Pietro G. di Summa, William Watfa

**Affiliations:** a Department of Plastic and Reconstructive Surgery, Saint George Hospital University Medical Center, Beirut, Lebanon; b Faculty of Medicine and Medical Sciences, University of Balamand, Tripoli, Lebanon; c School of Medicine and Medical Sciences, Holy Spirit University of Kaslik, P.O. Box 446, Jounieh, Lebanon; d Department of Plastic, Reconstructive, Aesthetic and Hand Surgery, University Hospital of Lausanne (CHUV), Lausanne, Switzerland; e Department of Plastic, Reconstructive, Aesthetic and Hand Surgery, Università Campus Biomedico, Rome, Italy

**Keywords:** Breast augmentation, Nipple-areola complex, Sensory change, Breast sensation, Implant plane, Incision approach

## Abstract

**Background:**

Breast augmentation is among the most frequently performed aesthetic procedures worldwide, offering high satisfaction levels but carrying potential sensory consequences that may affect function and quality of life. Preservation of nipple-areola complex and breast-skin sensation remains a key concern, given its significance for sexual and psychological functions. This systematic review aims to synthesize evidence regarding sensory outcomes following primary cosmetic breast augmentation.

**Methods:**

Following the PRISMA 2020 guidelines, a comprehensive search of PubMed/MEDLINE, Embase, Scopus, and Web of Science was conducted for studies published from 2000 to September 2026. Eligible studies included prospective or retrospective clinical investigations evaluating postoperative nipple-areola complex or breast-skin sensibility following primary breast augmentation, using objective measures (e.g., Semmes-Weinstein monofilaments, biothesiometry) or patient-reported outcomes. Non-English articles, reconstructive procedures, and secondary augmentations were excluded. Data were synthesized qualitatively due to the heterogeneity in study designs and outcome measures.

**Results:**

Twenty-six studies involving 9190 patients were included. Across studies, most sensory alterations were transient, with recovery typically occurring within six to twelve months. Periareolar incisions showed higher rates of early nipple-areola complex hypoesthesia, while inframammary and submuscular or subfascial placements were associated with fewer and milder changes. Implant volume demonstrated a limited but plausible mechanical effect, with larger devices exerting greater tension on superficial sensory branches. Discordance between objective and subjective assessments was noted, reflecting the nipple’s unique innervation pattern dominated by deep smooth-muscle fibers rather than superficial mechanoreceptors.

**Conclusion:**

Postoperative sensory alterations after breast augmentation are generally mild and reversible. Surgical planning that prioritizes incision placement, pocket selection, and implant dimensions within the anatomical limits of the skin envelope can minimize neural disruption. Future studies should use standardized sensory testing and patient-centered outcome measures to refine evidence-based strategies that preserve both aesthetic and functional integrity.

## Introduction and background

Breasts hold profound cultural, psychological, and aesthetic importance, often symbolizing femininity and sexuality.^1^ Their visual harmony and tactile qualities contribute significantly to self-image and interpersonal confidence.^2^ Beyond their aesthetic role, the breasts, and particularly the nipple-areola complex (NAC), serve essential sensory and functional purposes, influencing sexual satisfaction and overall quality of life.^3^ The NAC is a highly erogenous zone, richly innervated and uniquely responsive to tactile and thermal stimulation, making it integral to sexual arousal and gratification.^4^^,^^5^ Consequently, any alteration in breast sensation, whether congenital, pathological, or even iatrogenic in origin, may have considerable psychological and functional repercussions.^3^^,^^6^

Because the breast embodies both aesthetic and sensory dimensions, procedures that alter its form, such as augmentation, should be regarded as interventions affecting identity and overall well-being, not merely external appearance.^2^^,^^7^ The sensory integrity of the NAC remains closely tied to sexual and psychosocial health.^8^

In parallel with this psychosocial significance, the demand for breast aesthetic procedures has largely expanded. Breast augmentation is now among the most commonly performed cosmetic operations worldwide.^9^^,^^10^ According to the American Society of Plastic Surgeons, hundreds of thousands of augmentation surgeries are carried out annually in the United States, making it one of the most common cosmetic plastic surgery procedures.^10^^,^^11^

Despite high satisfaction rates and generally low complication profiles, preservation of NAC sensibility remains a core concern after augmentation.^12^ In fact, sensory outcomes are a key determinant of postoperative satisfaction, complementing measures such as contour and symmetry.^13^ Current augmentation practices involve a spectrum of surgical approaches, including different incision sites (most commonly inframammary and periareolar), implant planes (subglandular, subfascial, subpectoral, or dual-plane), and implant types (saline or cohesive silicone gel).^14^, ^15^, ^16^ Each of these technical variables can exert distinct effects on postoperative NAC sensibility.^14^^,^^17^ However, current findings are heterogeneous and often inconsistent across study designs, sensory testing methods, and follow-up durations.^17^, ^18^, ^19^

Beyond conventional primary implant-based augmentation, contemporary breast volumization increasingly includes alternative or adjunctive strategies such as mesh-assisted techniques, autologous fat grafting, composite augmentation, and augmentation-mastopexy, each of which may have distinct implications for breast and NAC sensibility. Mesh-based materials have primarily been developed to reinforce soft-tissue coverage and implant support, particularly in prepectoral reconstruction, although their specific effect on NAC sensory preservation remains insufficiently defined.^20^^,^^21^ Fat grafting, whether used alone or with implants, offers a distinct volumizing approach that may reduce the extent of prosthetic-pocket dissection, but current evidence does not establish a clear advantage for NAC sensory preservation.^11^^,^^22^, ^23^, ^24^ Likewise, mastopexy introduces additional factors such as NAC repositioning and periareolar tissue manipulation that may independently affect postoperative nipple sensibility.^25^^,^^26^ To maintain a clinically and methodologically coherent evidence base, the present review focused specifically on primary implant-based augmentation, allowing sensory outcomes to be examined in relation to implant-related surgical variables such as incision approach and pocket plane. Nonetheless, alternative and adjunctive augmentation strategies represent a relevant and expanding area for future comparative research into sensory preservation across the full spectrum of breast-volumizing procedures.

To address these gaps, the present review aims to consolidate and critically appraise the available anatomical and surgical knowledge concerning NAC, and to a broader extent, surrounding breast skin sensation after primary breast augmentation. Specifically, it seeks to synthesize findings across incision approaches and implant planes, and identify predictors of postoperative sensory outcomes. Ultimately, because nipple and breast sensibility are intimately linked to body image as well as sexual and psychosocial well-being, understanding and preserving this sensory dimension is vital to optimizing patient satisfaction and quality of life.^27^

## Methods

### Study design and guidelines

This study consists of a systematic review that was conducted in strict accordance with the Preferred Reporting Items for Systematic Reviews and Meta-Analyses (PRISMA) 2020 statement.^28^ The review aimed to synthesize and critically appraise all available evidence examining the effects of primary breast augmentation on NAC and breast sensation. The review protocol was not registered prospectively (e.g., with PROSPERO); this is acknowledged as a limitation under Strengths and Limitations.

### Search strategy

A comprehensive literature search was developed collaboratively by the study authors in consultation with an experienced medical librarian. The search was conducted across four electronic databases: PubMed/MEDLINE, Embase, Scopus, and Web of Science. Title/abstract and full-text screening were performed independently by two reviewers using a shared, dedicated systematic-review screening software (Rayyan). The complete electronic search strategy, including all search strings and field tags applied in each database, is provided in the accompanying Supplementary Material.

Search terms combined both controlled vocabulary and free-text keywords related to breast augmentation and sensory outcomes. The core conceptual strategy combined two term groups using Boolean AND/OR logic: (“breast augmentation” OR “breast enlargement” OR “augmentation mammoplasty”) AND (“breast sensation” OR “nipple sensation” OR “nipple sensitivity” OR “nipple-areola complex”), adapted to the specific syntax and controlled vocabulary of each database.

Searches were limited to English-language publications involving human subjects and published between January 2000 and September 2026 to capture modern implant technology and contemporary surgical techniques. The reference lists of all eligible full-text articles and relevant review papers were manually screened to identify additional studies not retrieved in the electronic search. Duplicate records across databases were identified and removed using reference-management software supplemented by manual review.

A schematic representation of the article identification and selection process was created using a PRISMA flow diagram (Fig. 1).

**Fig. 1:**
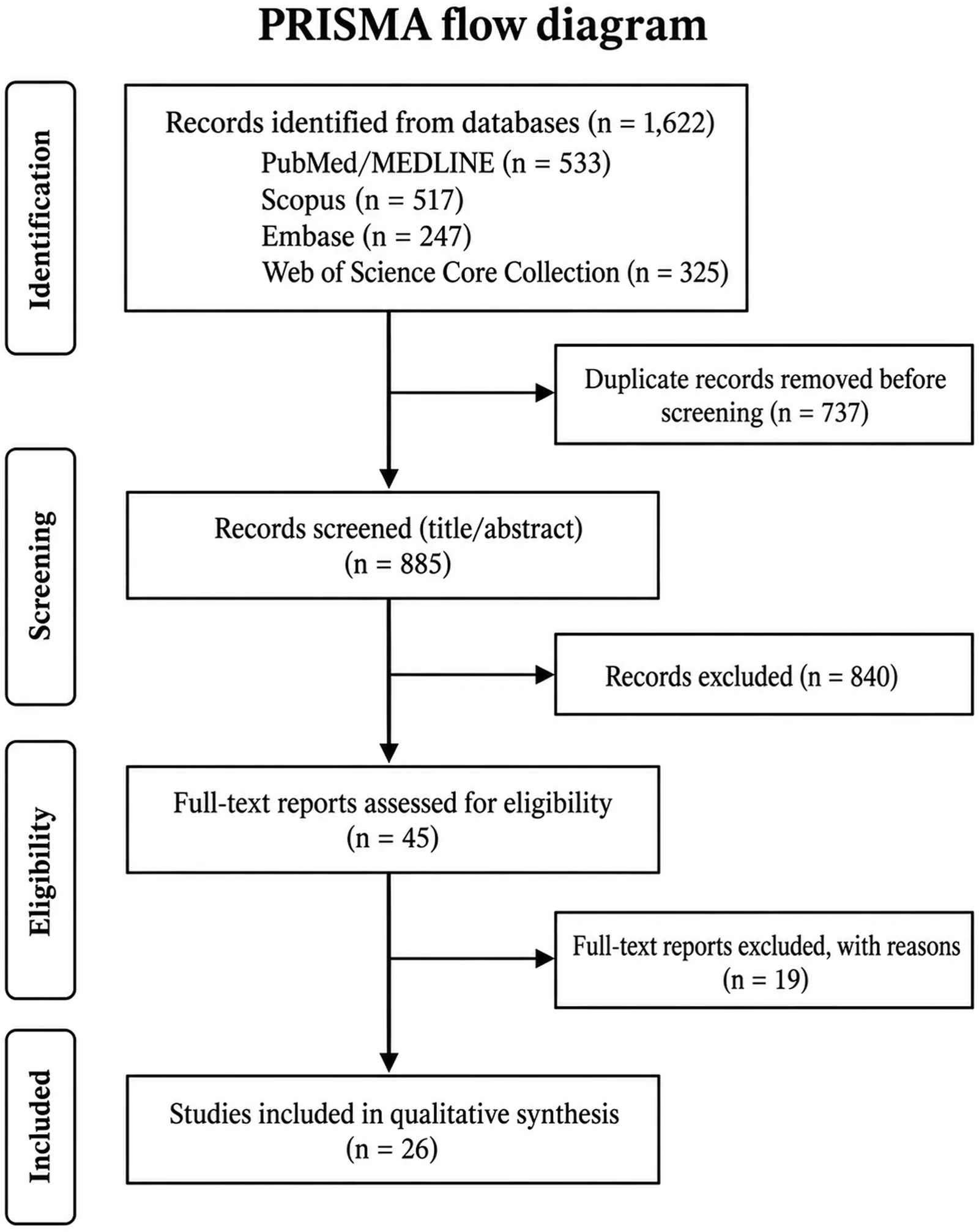
PRISMA Flow Diagram of Literature Search and Study Selection.

### Eligibility criteria

Inclusion and exclusion criteria were defined a priori to ensure methodological consistency during the screening process. Eligible studies were original clinical investigations, including randomized controlled trials, prospective cohort studies, and retrospective analyses that examined primary breast augmentation performed for cosmetic purposes. To be included, studies had to evaluate changes in breast or NAC sensation following surgery, using either objective sensory testing or validated patient-reported outcomes. Only articles published in English and available in full text were considered for review.

Studies were excluded if they involved breast reconstruction, breast reduction, mastopexy (breast lift), or gender-affirming augmentation procedures, as well as secondary or revision augmentations. Additional exclusion criteria comprised editorials, commentaries, conference abstracts, and narrative reviews. Research that did not assess sensory outcomes, studies conducted on animal models, and reports involving patients with bilateral breast cancer or prior thoracic surgery affecting sensory innervation were likewise excluded from analysis.

### Study selection process

Two independent reviewers screened all retrieved records in a two-stage process. Titles and abstracts were first reviewed to exclude clearly irrelevant studies. The remaining full texts were assessed in detail to confirm eligibility based on the inclusion and exclusion criteria. Any discrepancies between reviewers were resolved by discussion and consensus; a third reviewer arbitrated unresolved disagreements.

### Risk of bias and study quality assessment

We assessed the quality of the different studies by two reviewers, depending on the Newcastle-Ottawa Scale, with the sample selection, comparability and outcome.^29^ We paid particular attention to the rigor and standardization of sensory outcome, given the heterogeneity of its assessment. In this regard, large cohort studies such as those by Lund et al. and Wieslander relied primarily on narrative or non-standardized reporting of sensory outcomes, whereas smaller series more frequently used structured or objective sensory testing. Accordingly, rather than expanding the exclusion criteria, we used the Newcastle-Ottawa Scale scores descriptively to segregate and contextualize the results of the diverse studies. Disagreements in scoring were resolved by consensus. A summary of study quality assessment is provided in Supplementary Table S1.

### Data extraction

Data from all included studies were independently extracted by two reviewers using a standardized and pilot-tested Excel spreadsheet (Microsoft Corp.). For each study, detailed information was recorded on study characteristics, including the first author’s name, year of publication, country of origin, and study design. Patient demographics were documented as the total number of participants and mean age in years.

Surgical variables included the type of incision used with the number of patients per category, such as inframammary, periareolar, transareolar, transaxillary, or transumbilical. Implant-related parameters were comprehensively recorded, including brand or manufacturer, filling material (saline or silicone), surface texture (smooth, microtextured, or macrotextured, with corresponding patient distribution), and implant position within the breast pocket (subglandular, subfascial, subpectoral, or dual-plane, also with patient counts when reported).

When stated, sensory outcomes were initially stratified by incision type and implant plane to explore whether congruent patterns could be identified across the heterogeneous studies. Because several included studies combined different surgical approaches or implant characteristics within the same cohort and many did not provide subgroup-level data, stratification was performed only when numerical breakdowns were explicitly provided. In these cases, the number of postoperative dysesthesia or hypoesthesia events was extracted per reported incision type or implant plane, allowing aggregation of events only across studies that provided compatible denominators. Importantly, no assumptions or assertions were applied to studies missing subgroup data.

Whenever reported, sensory outcomes, such as the method of testing (e.g., objectively using Semmes-Weinstein monofilament and/or through patient-reported measures), timing of postoperative evaluation, and trends in sensation loss or recovery, were also extracted. We also recorded whether studies reported any formal sensory nerve coaptation or neurotization techniques aimed at supporting postoperative sensory recovery. If numerical data were unavailable but qualitative or descriptive information was provided, these findings were recorded narratively. All data discrepancies were resolved through consensus between the two reviewers to ensure accuracy and consistency across the dataset.

### Data synthesis and analysis

Because of considerable heterogeneity in study design, outcome definitions, follow-up intervals, and sensory assessment techniques, a formal meta-analysis was not feasible and results were synthesized qualitatively. Quantitative aggregation was prioritized for the two surgical variables most consistently and numerically reported across studies: implant plane and incision approach. When studies provided extractable numerators and denominators for these variables, we calculated descriptive aggregated proportions (i.e., simple sums of numerators and denominators across studies, not a formal meta-analytic pooled estimate) to describe the distribution and direction of reported sensory outcomes depending on incision approaches and implant planes. We used these quantitative summaries to compare patterns rather than to estimate the true incidence, given variability in outcome ascertainment. These aggregated frequencies do not account for between-study heterogeneity in sensory definitions, testing modalities, or follow-up duration, and are therefore reported strictly as descriptive summaries to illustrate directional trends rather than as pooled incidence estimates in the meta-analytic sense.

All remaining sensory findings were synthesized qualitatively, as the available literature frequently lacked standardized quantitative reporting or comparable baseline data. These included sensory testing modalities, magnitude of threshold change, recovery timelines, implant volume effects, and discrepancies between objective and subjective assessments. Such outcomes were analyzed narratively to account for methodological variability and to contextualize the quantitative findings. Patterns of postoperative NAC sensory change, including direction, extent, and temporal recovery, were therefore described according to the level of data consistency available for each variable.

## Results

The database search identified a total of 1622 records across the four databases searched: 533 from PubMed/MEDLINE, 517 from Scopus, 325 from Web of Science, and 247 from Embase. After removal of 737 duplicate entries, 885 unique records remained for screening. Full texts of potentially eligible articles were then assessed against the inclusion and exclusion criteria. Ultimately, 26 studies published between 2004 and 2026 met all inclusion criteria and were incorporated into the qualitative synthesis (Fig. 1).

### Study characteristics

The 26 included studies comprised a total of 9190 patients who underwent primary breast augmentation. Twelve were prospective and fourteen were retrospective in design, conducted across eighteen countries (Argentina, Sweden, Pakistan, the United States, Australia, Austria, China, Denmark, Italy, Germany, Brazil, Turkey, Egypt, Singapore, Chile, Norway, Colombia, and Costa Rica). Mean or median patient age ranged between 26 and 38 years when reported. Follow-up duration ranged from 2 weeks to 180 months, most commonly around 6 months. Details are presented in Table 1.

**Table 1:** Characteristics of the Included Studies.

Author and Year	Geographic Location	Study Design	n	Age (years)	Follow-Up (months)	Incision Type	Implant Type and Texture	Implant Plane	Implant Volume (mL)	Sensory Assessment Type	Sensory Outcomes
Rancati et al., 2023	Argentina	Prospective cohort	50	28.4	6	Inframammary medial (n = 25; 50%) Inframammary laterally displaced (n = 25; 50%)	Silicone, with smooth round texture (n = 50)	Subglandular (n = 50)	292.8 for the conventional medial cohort and 283 for the laterally displaced cohort	Objective testing with Semmes-Weinstein monofilaments across the NAC and four breast quadrants.	Laterally displaced inframammary incisions preserved normal sensation in all quadrants and NAC at all time points. Conventional medial incisions produced sensory reduction that improved by Day 30 but remained below preoperative levels at 6 months.
Wieslander, 2020	Sweden	Retrospective cohort	1310	NR	4, 60, 120, and 180	Transaxillary (n = 1310)	Silicone with fine and cohesive texture (n = 1310)	Submuscular (n = 1310)	350	Subjective clinical and patient-reported follow-up.	Transient changes in sensation were common early postoperatively, with full normalization of NAC sensation within 2 - 6 months. No persistent sensory loss was reported.
Ahmad, 2017	Pakistan	Retrospective case series	43 of which 3 were fat-transfer	27.51	NR	Inframammary (n = 30); Periareolar (n = 6); Transumbilical (n = 4)	Silicone (n = 30); Saline (n = 13)	Subfascial	279 for the inframammary approach group (n = 30); 292 for the periareolar approach group (n = 6); 278 for the transumbilical approach group (n = 4)	Subjective clinical and patient-reported follow-up.	Transient changes in sensation in 6 patients (13.8%), mainly involving the breast skin and NAC, predominantly in the periareolar group (n = 4). All recovered normal sensation during follow-up.
Lund et al., 2016	USA	Prospective multicenter cohort	4927	Median of 36 (18 - 82)	1, 6 and up to 120 (mean of 49.2)	Inframammary (n = 4621); Periareolar (n = 306)	Silicone, with Biocell textured surface (n = 4927)	Dual-plane (partial submuscular) (n = 4353); Submuscular (complete) (n = 114); Subglandular (n = 456); Subfascial (n = 4)	Ranging from 165 to 740, most commonly around 400	Subjective clinical and patient-reported follow-up.	Transient changes in sensation in 19 patients (0.4%) within the inframammary group; none in periareolar group. Skin sensation changes in 5 patients (0.1%); all mild and mostly resolved. Larger implants were associated with more sensory alterations. No lasting sensory deficits reported.
Brown et al., 2016	Australia	Prospective cohort	162	30.87	3	Inframammary (n = 162)	Silicone (n = 162)	Subfascial (n = 162)	472	Objective testing with Semmes-Weinstein monofilaments across the NAC and four breast quadrants.	Mild transient changes in sensation in 32 patients (19.8%), most frequent in the lower-outer quadrant (16.15%) and NAC (4%). All changes were mild and transient, with majority achieving full recovery by follow-up.
Zhu et al., 2015	China	Retrospective	15	35.4	4 - 8 (mean of 6.7)	Periareolar (n = 15)	Silicone, with round texture (n = 15)	Submuscular (n = 15)	248	Subjective clinical and patient-reported follow-up.	Transient changes in sensation in the early postoperative period, affecting the nipple and breast skin, with no permanent sensory loss at 4 - 8 months follow-up.
Mohmand and Ahmad, 2013	Pakistan	Prospective observational	32	30.7	6	Periareolar (n = 32)	Silicone (n = 32)	Subglandular (n = 32)	308, with a range of 175 - 435	Subjective clinical and patient-reported follow-up.	Transient changes in sensation were observed in 7 patients (21.9%) at 3 months, improving to 2 patients (6.3%) at 6 months.
von Sperling et al., 2011	Denmark	Retrospective	95	34.2	Mean of 31.8	Inframammary (n = 95)	Silicone, with round cohesive texture (n = 95)	Subglandular (n = 66); Submuscular (n = 28)	NR	Subjective through patient-filled questionnaire.	Long-term persistent changes in sensation in 72 patients (75.8%), including decreased sensation in 66 (69.5%) and increased sensation in 30 (31.6%). Most changes were mild to moderate, with overlapping areas of sensory alteration and pain.
Araco et al., 2011	Italy	Retrospective comparative	1222	31	6	Periareolar (n = 189); Inframammary (n = 1033)	NR	Subglandular (n = 611); Submuscular (n = 611)	344	Subjective clinical and patient-reported follow-up.	Changes in sensation of the NAC occurred in 55 patients (4.5%), more with the periareolar than with the inframammary incision.
Niechajev et al., 2007	Sweden	Prospective	80	Median of 28	(48 - 62) median of 60	Periareolar (n = 10); Inframammary (n = 69); Transaxillary (n = 1)	Silicone, textured cohesive (n = 80)	Subglandular (n = 58); Submuscular (n = 22)	310	Objective through gentle finger-touch testing of four quadrants.	Change in sensation of breast skin in 38 (26%) of augmented breasts, predominantly as mild decline or paresthesia localized to the triangular area above or below the incision. This decrease occurred in 25% of breasts adjacent to the incision, particularly following periareolar access. No relationship between implant position or volume and changes in skin or nipple sensation. 17 cases had increased sensitivity, 16 out of which in the subglandular pocket.
Pitanguy et al., 2007	Brazil	Prospective clinical	29 (56 breasts)	33	6	Periareolar (n = 31 breasts, 55.3%); Inframammary (n = 17 breasts, 30.3%); Transareolar (n = 8 breasts, 14.3%)	Silicone (n = 28)	Subglandular (n = 29)	230	Objective and subjective assessment using Semmes-Weinstein monofilaments and patient questionnaires.	Changes in sensation were observed in 21 breasts (37.5%) subjectively and in 18 breasts (32.1%) objectively, most affecting the inferior areola. No difference was found between periareolar and inframammary approaches; the transareolar group (n = 8 breasts) was too small for valid statistical comparison. Relative implant volume (implant-to-breast-volume ratio) was independently associated with sensibility alterations (p < 0.05).
Sevin et al., 2006	Turkey	Retrospective case series	210 breasts	NR	(60 - 120) mean of 96	Periareolar (n = 168); Inframammary (n = 42)	Silicone, round (n = 210)	Submuscular (n = 210)	NR	Subjective clinical and patient-reported follow-up.	Transient decrease in sensation in 5 augmented breasts (2%). All changes were mild and limited to the nipple or adjacent breast skin.
Okwueze et al., 2006	USA	Prospective clinical trial	33 out of which 24 were followed-up at 6 months	33	6	Inframammary (n = 11); Periareolar (n = 13)	Saline (n = 24)	Submuscular (n = 24)	360	Objective and subjective assessment using a pressure-specified sensory device and patient survey.	Transient changes in sensation with increased thresholds at 2 - 4 weeks; by 6 months most regions returned to baseline, with residual elevation in the inferior breast and areola. The periareolar approach showed less change in sensation at the inferior breast than the inframammary approach; NAC sensitivity was similar between approaches. The size of the implant had no significant impact on breast sensitivity.
Mofid et al., 2006	USA	Retrospective comparative	29 (9 controls)	33	13.4 (mean)	Inframammary (n = 13); Periareolar (n = 7)	NR	Subglandular and Submuscular (no subdivision numbers were mentioned)	375	Objective testing with a Pressure-Specified Sensory Device assessing one-point static and moving thresholds of the NAC, upper and lower nipple and areola.	Long-term changes in sensation postoperatively, with thresholds nearly 10 times higher than in unoperated controls. No difference was detected between periareolar and inframammary incisions. Larger implant volumes were associated with greater changes in sensation. No improvement in sensation was noted beyond 6 months.
Stoff-Khalili et al., 2004	Germany	Retrospective comparative	328	34	43.2	Periareolar (n = 328)	Silicone, smooth and textured surfaces	Subglandular (n = 105); Submuscular (n = 154); Subfascial (n = 69)	325	Subjective clinical and patient-reported follow-up.	Changes in nipple sensation in 12 patients (11.4%) with subglandular implants, 7 (4.5%) with subpectoral, and 3 (4%) with subfascial placement. Subfascial augmentation markedly reduced sensory changes compared with subglandular placement while maintaining low complication rates and improved comfort. Larger implants were associated with more sensory alterations.
Kompatscher et al., 2004	Austria	Retrospective	18	29	24	Transareolar (n = 18)	Silicone textured (n = 18)	Submuscular (n = 18)	Ranging from 115 to 325	Objective and subjective testing with Semmes-Weinstein monofilaments, biothesiometer, and thermal discrimination across nine breast coordinates (objective), supplemented by patient-reported sensation and nipple erectility (subjective).	Subjectively, 16 patients (89%) reported normal sensation 2 years post-op, though objective testing showed mild to moderate loss of sensation and elevated vibration thresholds across all regions. Nipple erectility was preserved.
Banbury et al., 2004	USA	Prospective	47	34	3 and 6	NR	Saline (n = 47)	Submuscular (n = 47)	335	Objective and subjective testing with Semmes-Weinstein monofilaments and biothesiometer at nine breast coordinates (objective), supplemented by patient-reported sensation at 6 months (subjective).	Decrease in pressure and vibration sensation noted at 3 months post-op, returning to baseline by 6 months. At 6 months, 9 patients (20%) reported decreased and 9 others (20%) increased sensation. No correlation between subjective and objective measures. No persistent sensory loss detected.
Ashraf et al., 2021	Egypt	Retrospective comparative	18 (36 breasts)	29	2, 6, and 12 weeks	Inframammary (n = 18)	Silicone, round smooth gel-filled (n = 18)	Subglandular (n = 9 patients/18 breasts); Subfascial (n = 9 patients/18 breasts)	Ranging from 235 to 395	Objective testing with Semmes-Weinstein monofilaments across the NAC (nipple and four areolar quadrants) preoperatively and at 2, 6 and 12 weeks postoperatively.	At 12 weeks, partial NAC sensory alteration occurred in 5% of subfascial breasts versus 28% of subglandular breasts, most affecting the inferior and lateral areola. No correlation with implant volume was found.
Üstün et al., 2025	Turkey	Retrospective comparative with unoperated control group	55 (110 breasts); 20 controls (40 breasts)	35.7	Mean 1562 days (range 397–3514)	Inframammary (n = 55)	Silicone, round (83%) and anatomic (17%)	Dual-plane 1 (n = 40 breasts); Dual-plane 2/3 (n = 34 breasts); Subglandular (n = 36 breasts)	NR	Objective testing with Semmes-Weinstein monofilaments at 9 points per breast (central and peripheral composite scores), compared with an unoperated control group.	Sensation was significantly worse in the operated group than in controls, centrally, peripherally, and overall (all p < 0.001). Subglandular placement preserved central sensation better than dual-plane 2/3 (p = 0.04) but resulted in significantly worse peripheral sensation than both dual-plane groups (p < 0.001). Implant size, type, smoking, and systemic disease were not significantly associated with sensation.
Shi et al., 2015	China	Retrospective comparative	144 (39 subglandular; 105 subpectoral)	38.1 (subglandular); 31.2 (subpectoral)	Mean 32.5 months (subglandular); 31.7 months (subpectoral)	Axillary or periareolar (not stratified by plane)	Silicone, cohesive gel, textured (n = 144)	Subglandular (n = 39); Subpectoral (n = 105)	≈202 (subglandular); ≈200 (subpectoral)	Subjective clinical follow-up for complications, including nipple-areola numbness, recorded per implant.	Nipple-areola numbness occurred in 5 of 70 subglandular implants (7.1%) versus 0 of 178 subpectoral implants (0%; p < 0.05). Subglandular placement had a shorter recovery period; overall complication and satisfaction rates were similar between groups.
Randquist et al., 2018	Singapore (Southeast Asian patients)	Retrospective chart review with patient-reported outcomes	22 (of 31 treated patients)	35.1	11 months to 5.5 years	Inframammary (n = 22)	Silicone, anatomical form-stable (n = 22)	Dual-plane (n = 22)	NR	Patient-reported complications (BREAST-Q, POSAS) rather than structured sensory testing.	Among the 22-patient analyzed cohort, patient-reported complaints included sensory loss at the nipple (n = 2) and around the nipple (n = 3); no reoperations were required. High long-term BREAST-Q satisfaction scores were reported.
Prado et al., 2008	Chile	Prospective cohort	28	NR	13 months	Caudal-periareolar (n = 28)	Silicone, textured (McGhan Style 120) (n = 28)	Retromuscular/submuscular (n = 28)	NR	Objective faradic electrical perception-threshold testing of the NAC (electromyographic device), preoperative and 13 months postoperative.	All patients had a higher faradic perception threshold at 13 months versus preoperative baseline, and 3 of 28 (10.7%) had lost NAC sensibility entirely; preoperative thresholds in hypoplastic breasts did not differ from normal volunteers.
Kaasa et al., 2010†	Norway	Prospective RCT-derived cohort, 4-year follow-up	116 (of 175 original cohort)	33	4 years	NR	NR	NR	NR	Subjective, patient-reported pain and sensory-change questionnaire (hyperesthesia/hypoesthesia) at 6 weeks, 1 year, and 4 years.	Hyperesthesia declined from 46% at 1 year to 32% at 4 years; hypoesthesia was stable (47% to 51%). Concomitant pain and hyperesthesia at 6 weeks/1 year strongly predicted persistent pain at 4 years.
Balaguera Herrera et al., 2026‡	Colombia	Prospective cohort (novel technique)	64	26 (range 20–45)	1 year (sensation assessed at 2 months)	Periareolar (n = 64)	Silicone, round (Motiva) (n = 64)	Submuscular, muscle-glandular-preserving “preservation” technique (n = 64)	275–375	Tactile discrimination test (method not further specified), assessed 2 months postoperatively.	Sensation was normal in both breasts in all patients at 2 months. ‡Novel single-arm technique series without a comparator group.
Chacon-Quiros et al., 2026‡	Costa Rica	Prospective cohort (novel minimally invasive technique)	100	28.6 (range 18–44)	3 years	Axillary (n = 100)	Silicone, biconvex-shaped (Motiva Mia Femtech) (n = 100)	Prepectoral (n = 100)	Mean 148 (range 100–190)	Patient-reported complication surveillance at scheduled visits; no structured NAC sensory-testing instrument used.	No patient reported loss of nipple or breast sensation through 3 years of follow-up. ‡Novel single-arm technique series without a comparator group.
Stokes, 2004	USA	Retrospective questionnaire study	13 (26 breasts)	NR	NR	NR	Saline (implied by title; not otherwise specified)	NR	NR	Subjective, patient-reported questionnaire.	Of 26 augmented breasts, sensation was diminished in 5 (19.2%), increased in 5 (19.2%), and similar to preoperative baseline in 16 (61.5%).

NR = Not reported; n = number of patients unless otherwise specified in a given cell (e.g., breasts, implants, controls, or survey respondents, as noted); USA = United States of America.

### Study quality assessment

Study quality was assessed independently by two reviewers using the Newcastle–Ottawa Scale (maximum score of 9), as summarized in Supplementary Table S1. Among the 26 studies for which sufficient methodological information was available for formal assessment, total scores ranged from 3 to 8. Ten studies (38.5%) scored 7 or higher and were considered of high methodological quality. These studies generally incorporated objective or structured sensory assessment, appropriate comparator groups, direct comparisons between incision types or implant planes, or a combination of these methodological strengths. Eight studies (30.8%) scored 5–6 and were considered of moderate quality, most commonly reflecting adequate cohort selection and follow-up but limitations related to subjective or non-standardized sensory assessment, or limited control for comparability. The remaining eight studies (30.8%) scored 4 or lower and were considered of lower methodological quality; these were predominantly small or single-arm observational series without comparator groups and frequently relied on patient-reported sensory outcome ascertainment rather than validated objective testing. Notably, the two numerically largest cohorts, by Lund et al. and Wieslander, received moderate-quality scores of 6 and 5, respectively, despite their large sample sizes, largely because sensory outcomes were captured through case report forms or narrative clinical assessment rather than standardized instrument-based testing.

### Surgical and implant parameters

Across the 9190 patients included, and specifically among the 8868 patients in whom the surgical incision was specified, inframammary incisions predominated (n = 6230, 70.3%), followed by transaxillary (n = 1411, 15.9%) and periareolar/transareolar approaches (n = 1223, 13.8%), whereas transumbilical access was rare (n = 4, 0.05%). Silicone cohesive gel implants predominated across the studies (reported in approximately 98% of series specifying implant type), while saline implants were reported mainly in USA studies and in one mixed cohort by Ahmad.^30^ Implant placement was mainly submuscular or dual-plane (n = 6883, 75.8%), while subglandular (n = 1903, 21.0%) and subfascial (n = 295, 3.2%) placements were less common. Reported implant volumes ranged between 100 and 740 mL, most clustering around 300–400 mL.

### Sensory assessment and general outcomes

Sensation was evaluated using both objective and subjective approaches across the included studies. Objective modalities included Semmes-Weinstein monofilament testing, pressure-specified sensory devices, biothesiometry, and thermal discrimination, while subjective assessments relied on clinical evaluation and patient-reported outcomes, as well as standardized questionnaires. Semmes-Weinstein monofilaments are graded nylon filaments that apply standardized pressure thresholds to quantify cutaneous sensory detection.^31^ Pressure-specified sensory devices quantify pressure perception and two-point discrimination by measuring the minimum pressure required for sensory detection. Biothesiometry assesses vibration perception thresholds to evaluate large-fiber sensory nerve function. Thermal discrimination testing evaluates the ability to detect temperature differences, reflecting small-fiber sensory integrity.^32^

Most studies examined both the NAC and multiple breast quadrants to capture regional sensory variation. Transient postoperative sensory alteration was the most consistent observation. Objective monofilament testing in the cohort by Brown et al. demonstrated mild, temporary threshold elevations, with most patients recovering preoperative levels of sensation by 12 weeks.^33^ Wieslander similarly noted early numbness and tingling with full normalization of NAC sensation between two and six months.^34^ The large multicenter analysis by Lund et al. reported NAC sensory change in only 0.4% of inframammary cases and none of the periareolar group, with minor cutaneous alterations (0.1%) that were self-limiting.^35^ Smaller series by Zhu et al. and Mohmand and Ahmad also similarly showed comparable transient hypoesthesia of the nipple or breast skin that resolved entirely by the end of follow-up.^36^^,^^37^

### Incision approach and sensory outcomes

When aggregated across comparable studies, and accounting for substantial variability in design and reporting, periareolar incisions resulted in higher rates of extractable sensory alterations than inframammary approaches. Across studies that provided explicit numerators and denominators, periareolar incisions showed postoperative sensory changes in approximately 8.1% of patients (n = 73/904), whereas inframammary incisions demonstrated a descriptive aggregated rate of roughly 2.1% (n = 124/5905). These values changed widely according to sensory assessment method, ranging from very low rates (as low as 0.4%) in large narrative-based cohorts to higher proportions (up to 20%) in smaller studies that used structured or objective testing.^30^^,^^35^, ^36^, ^37^, ^38^, ^39^, ^40^ As outlined in the Methods, these frequencies are descriptive cross-study summaries and are interpreted primarily for their directional patterns. To note that, when the analysis was restricted to studies with defined sensory methods and a higher total score on the Newcastle-Ottawa Scale, the directional relationship between incision approach and reported sensory alteration remained unchanged. The transaxillary approach, reported in isolation and without comparator groups, showed a low rate of sensory loss, approximately at 0.7% (n = 9/1332). In general, the distribution of sensory change differed by incision site: periareolar access more frequently affected the NAC region, whereas inframammary incisions, particularly medial placements, were more often associated with inferior-pole or lower-quadrant skin hypoesthesia.^36^^,^^38^^,^^40^ Both patterns were mostly transient and resolved within a few months.

Notably, Araco et al. analyzed a sample of 1222 patients undergoing either periareolar or inframammary augmentation using consistent clinical sensory assessment and direct incision-group comparison, and reported NAC sensory alteration in 4.5% of cases, with a significantly higher proportion in the periareolar group.^41^ Mofid et al. objectively assessed 20 augmented patients using a pressure-specified sensory device and found no statistically significant difference in nipple-areola thresholds between inframammary and periareolar incisions, although both groups showed overall sensitivity reduction compared to a separate unoperated control group, but the lack of preoperative sensibility data in the augmented patients was an acknowledged key limitation preventing the determination of exact postoperative change.^17^ According to Pitanguy et al., postoperative monofilament testing across nine breast regions revealed no difference in either objective or subjective sensory loss between incision types, with alterations more closely associated with implant volume than incision site.^42^ In the Pakistani series by Ahmad, transient sensory changes occurred in 6 patients (13.8%), predominantly in the periareolar group, all of which resolved during follow-up.^30^ In contrast, Prado et al. found persistent objective NAC threshold elevation 13 months after caudal-periareolar augmentation, including complete sensory loss in 3 of 28 patients.^43^

Lund et al., who compared inframammary and periareolar augmentations, concluded that nipple sensation change occurred in 0.3% - 0.4% of inframammary cases at 6 months and 10 years, with no sensory change reported in the periareolar cohort.^35^ Similarly, Wieslander, reporting on a transaxillary cohort, described predominantly transient sensory symptoms with full recovery by 2–6 months, based on clinical follow-up rather than formal sensory measurement.^34^ Randquist et al. also reported only limited long-term sensory complaints after inframammary dual-plane augmentation, with nipple sensory loss in 2 patients and perinipple sensory loss in 3.^44^

Also, Niechajev et al. documented decreased skin sensitivity in 26% of augmented breasts, predominantly mild and limited to areas adjacent to the incision, with 80% of nipples retaining normal sensation and 10% exhibiting increased sensitivity.^39^ Okwueze et al. reported comparable overall sensory patterns between periareolar and inframammary incisions; however, at 6 months, the inferior breast region showed significantly higher sensitivity thresholds following inframammary incision.^40^

Finally, Rancati et al. reported that laterally displaced inframammary incisions preserved normal NAC and quadrant sensation at all postoperative intervals, whereas conventional medial inframammary incisions showed uniform transient hypoesthesia up to 6 months after surgery.^38^

### Implant plane and sensory outcomes

As for the implant plane, sensory recovery varied according to the depth of placement. When restricting the analysis to studies that reported extractable numerators and denominators, submuscular pockets demonstrated the lowest aggregated descriptive rate of sensory alteration (1.61%; n = 106/6596), followed by subfascial placement (7.87%; n = 10/127). In contrast, subglandular insertion showed a substantially higher aggregated descriptive rate of postoperative sensory change (11%; n = 81/734).

In particular, Stoff-Khalili et al. reported nipple sensation changes in 11.4% of subglandular cases, 4.5% of subpectoral, and 4% of subfascial augmentations.^45^ Ashraf et al. similarly compared subglandular and subfascial placement through a uniformly inframammary incision, and found partial NAC sensory alteration in 28% of subglandular breasts compared with 5% of subfascial breasts at 12 weeks.^46^ Shi et al., comparing subglandular and subpectoral placement, recorded nipple-areola numbness as a clinically observed complication in 7.1% of subglandular implants versus none in the subpectoral group.^47^ In contrast, Niechajev et al. found no statistically significant association between implant plane and sensory change, and the large multicenter cohort by Lund et al. likewise reported no measurable relationship between implant position and postoperative nipple or skin sensitivity.^35^^,^^39^

On the other hand, Pitanguy et al. reported decreased NAC sensation in 62% of subglandular cases, predominantly at the lower areolar region, whereas Brown et al. documented mild and transient sensory alterations in 19.8% of subfascial cases, with normalization by the end of follow-up.^33^^,^^42^ Üstün et al., using a 9-point Semmes-Weinstein protocol, found that subglandular placement was associated with better central NAC sensation but worse peripheral sensation than dual-plane placement, suggesting a region-dependent effect of implant plane.^48^

### Additional sensory findings across studies

Additional studies provided complementary insights into postoperative sensory variation. Banbury et al. and Okwueze et al. demonstrated transient threshold elevations on pressure and vibration testing that returned to baseline by six months.^40^^,^^49^ In contrast, Mofid et al. identified persistently higher NAC thresholds compared with unoperated controls, especially in patients with larger implant volumes.^17^ Lund et al. similarly reported that increased implant size correlated with a higher incidence of temporary sensory alterations, while Okwueze et al. found no significant effect of implant size on postoperative breast sensitivity.^35^^,^^40^

Kompatscher et al. documented mild-to-moderate losses on objective biothesiometry, noting an increase of one to two Semmes-Weinstein monofilament levels despite 89% of patients reporting normal subjective sensation.^50^ These postoperative threshold shifts remained within the lower sensitivity range, as Semmes-Weinstein values of 3.84 or higher represent diminished protective sensation. Thresholds below this cut-off, even when elevated relative to baseline, fall within the range of normal variation observed in non-operated breasts.^13^

Isolated long-term alterations were described by von Sperling et al., who found persistent sensory changes in 75.8% of patients, frequently accompanied by discomfort or pain.^51^ Additionally, prospective and retrospective series by Niechajev et al., Sevin et al., and Stokes similarly reported localized and generally mild sensory alterations near incision sites, with complete or near-complete recovery over time.^39^^,^^52^^,^^53^

Finally, none of the studies reported any formal sensory nerve coaptation or neurotization techniques aimed at supporting sensory recovery.

## Discussion

This systematic review synthesizes clinical evidence on NAC and breast skin sensibility following primary cosmetic breast augmentation, with particular attention to incision approach, implant plane, and selected device characteristics. Our aim was to delineate the extent and timeline of sensory change and to appraise the methodological features that shape interpretation and clinical applicability. Across heterogeneous designs and testing protocols, the included studies converge on a coherent signal that facilitates practical counseling and operative planning, taking into account the need for standardized outcome measurements.

Across the 26 included studies, the dominant postoperative pattern reflected a transient rather than permanent alteration in breast and NAC, mostly confined to the first 6 to 12 months after surgery. Studies using objective sensory modalities, including Semmes-Weinstein monofilaments, pressure-specified sensory devices, and vibrotactile testing, demonstrated an early elevation in detection thresholds that progressively returned to baseline on follow-up.^33^^,^^38^^,^^50^ Similar findings were observed in patient-reported outcomes, with temporary numbness or tingling followed by recovery nearly to their preoperative levels.^30^^,^^34^^,^^37^^,^^52^ This pattern aligns with the interpretation that the peripheral nerve response to the limited surgical trauma involves physiological axonal regeneration and collateral reinnervation within the cutaneous plexus supplying the NAC.^54^ Nevertheless, long-term follow-up suggests that sensory disturbances may persist more often than shorter-term studies imply, particularly when symptoms are assessed systematically over several years.^55^

### Incision approach and sensory outcomes

Anatomically, these transient sensory changes can be explained by the distribution of the intercostal nerve branches, mainly the fourth intercostal nerve, which provides the dominant afferent supply to the NAC via its anterior and lateral cutaneous branches.^56^ These branches penetrate the deep fascia, traverse the glandular parenchyma, and ramify within the subdermal plexus encircling the areola, making them particularly susceptible to surgical manipulation.^57^^,^^58^ In this review, inframammary and periareolar incisions represented the most frequently compared access routes, together constituting the majority of reported cases. When these descriptive frequencies were aggregated across studies, periareolar access demonstrated a higher rate of early NAC hypoesthesia, whereas inframammary approaches more often produced inferior pole or lower quadrant skin hypoesthesia.^30^^,^^35^^,^^38^^,^^40^, ^41^, ^42^, ^43^ Both alterations were predominantly transient, with a near-complete recovery within 3 to 6 months postoperatively.^30^^,^^35^, ^36^, ^37^

This distribution aligns with the anatomical course of the intercostal nerve branches supplying the NAC. The latter, receives its dominant innervation from the anterior and lateral cutaneous branches of the third to fifth intercostal nerves, which course superficially within the subdermal plexus before converging toward the areola. A periareolar or radial incision, running transversely through this region, carries a higher risk of traversing these terminal branches and the subareolar plexus itself, thereby increasing the likelihood of temporary axonal disruption and early postoperative hypoesthesia.^41^^,^^58^^,^^59^ Notably, Araco et al., combining a large sample size with direct incision-group comparison, attributed the higher incidence of NAC sensory changes following periareolar access to superficial nerve fiber disruption and to restricted visualization that may compromise preservation of the lateral neurovascular structures.^41^ In contrast, the inframammary incision lies anatomically farther from the subareolar neural plexus and is therefore less likely to disrupt the primary afferents supplying the NAC. Patients receiving inframammary fold incisions demonstrate lower rates of sensory alterations compared to periareolar or radial access, suggesting a relative advantage in preserving nipple sensibility.^40^^,^^59^ However, subtle threshold elevations are sometimes noted along the inferior breast following inframammary access, reflecting localized and transient cutaneous hypoesthesia rather than true denervation.^40^^,^^59^

The transaxillary incision, on the other hand, was not amenable to direct comparison because it was evaluated in isolation, although it demonstrated low rates of sensory loss. As reported by Wieslander, NAC sensation returned to normal within two to six months postoperatively, but the absence of objective sensory testing and direct comparative data precludes drawing firm, evidence-based conclusions regarding its relative impact on postoperative sensibility compared with other incision types.^34^

Clinically, minimizing postoperative NAC sensory deficits relies more on meticulous surgical planning than on interventions after surgery, which in part relies on incisions that spare the subareolar neural plexus whenever possible.^59^^,^^60^

### Implant plane and sensory outcomes

The relationship between implant plane and postoperative sensibility is largely determined by the proximity of dissection to the major sensory pathways supplying the NAC. As previously mentioned, the NAC is innervated predominantly by the lateral and anterior cutaneous branches of the third to fifth intercostal nerves, which course along the chest wall, cross the pectoral fascia, and then within or just beneath the superficial glandular/fascial plane before forming the subareolar plexus.^61^ Accordingly, subglandular augmentation, performed directly beneath the glandular parenchyma and superficial fascia, places these terminal nerve branches at greatest risk of traction or partial axonal injury, consistent with the higher incidence of sensory alterations observed in this plane. Notably, however, Üstün et al. found better central NAC sensation with subglandular placement than with dual-plane 2/3, despite poorer peripheral sensation than with either dual-plane group, indicating that plane-related sensory effects may vary according to the specific breast region assessed. Nevertheless, deeper implant placement may generally reduce manipulation of the neural arborization supplying the nipple and surrounding skin.^62^

Submuscular pockets, in particular, are created deep to pectoralis major and usually preserve the superficial fascial plane and subareolar plexus, so sensory alterations are more likely mediated by tissue stretch (implant volume, pocket expansion) than by direct transection of the intercostal branches.^17^^,^^63^^,^^64^ Subfascial dissection theoretically occupies an intermediate position: it maintains a gland-implant interface but involves elevating the pectoral fascia itself, where lateral intercostal branches and their perforating branches may run in close contact.^64^^,^^65^ This could explain why subfascial placements tend to show lower rates of sensory change than subglandular pockets^46^ but do not completely abolish the risk of NAC or skin hypoesthesia. Fig. 2 depicts the principal implant planes together with the course of the third to fifth intercostal nerves and their terminal branches supplying the NAC, illustrating the differing degrees of neural vulnerability across planes.

**Fig. 2:**
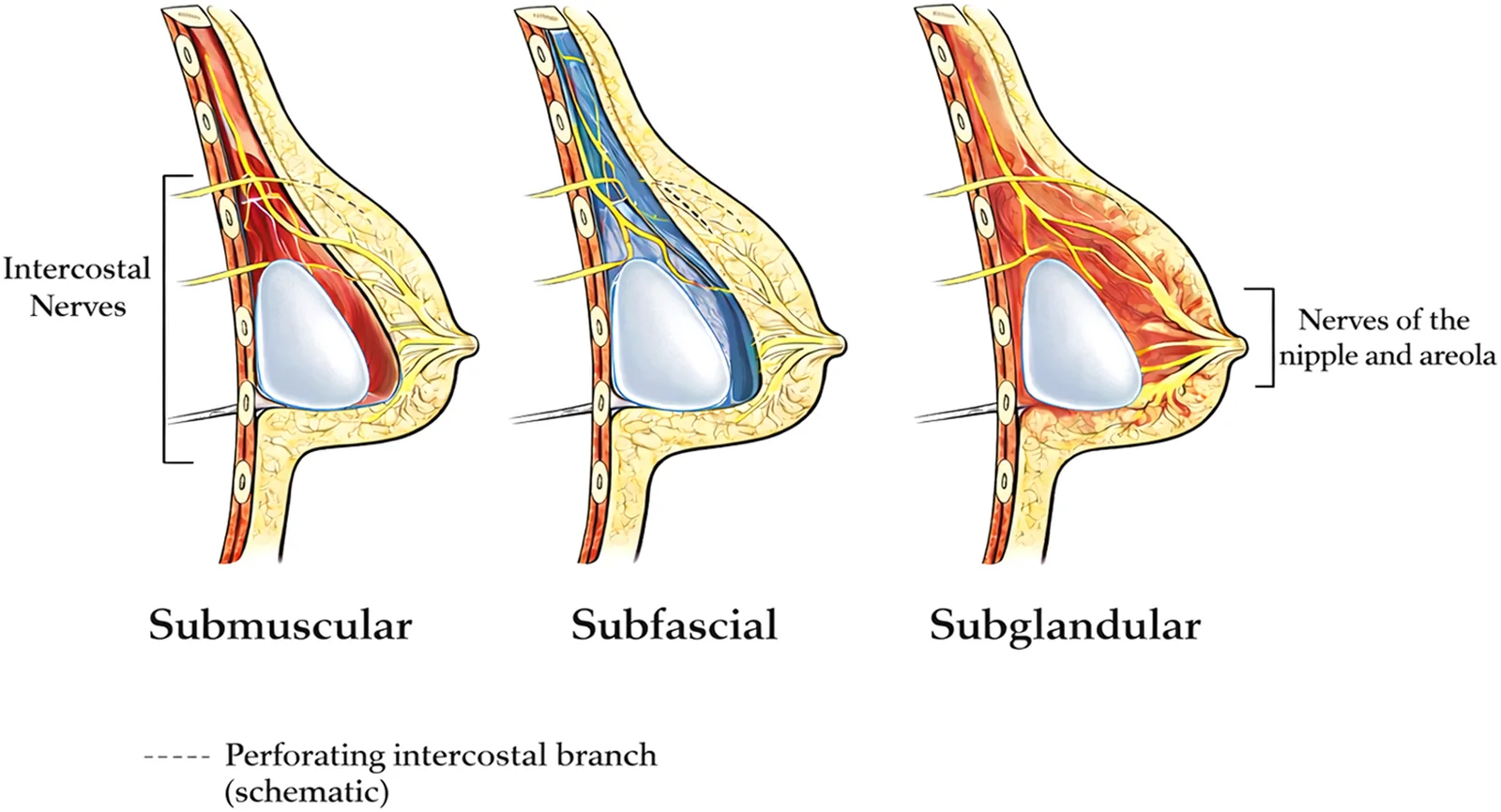
Cross-sectional graphic of the breast demonstrating subfascial, submuscular, and subglandular implant planes with the course of the lateral intercostal sensory branches shown schematically. The figure reflects the relative proximity of each implant plane to the superficial neural pathways supplying the nipple-areola complex and demonstrates the potential course of intercostal nerves along the pectoral fascia. Because the subglandular pocket is created immediately superficial to the pectoral fascia and deep to the glandular parenchyma, the terminal perforating intercostal nerve branches, as shown in the schematic representation of the subglandular pocket, are more susceptible to traction and partial axonal injury, whereas subfascial, and to a greater extent submuscular, placements preserve the superficial fascial plane and minimize disruption of the neural arborization supplying the nipple-areola complex.

Nevertheless, heterogeneity in pocket dissection techniques, implant volume, and reporting methods across studies limits the generalizability of these findings. The available evidence supports a trend toward lower and more transient sensory disturbances with submuscular and subfascial placements,^33^^,^^45^, ^46^, ^47^ but standardized quantitative assessments are required to delineate their comparative advantages.

### Additional sensory findings across studies

The effect of implant volume on postoperative sensation appears limited but mechanistically reasonable. Mofid et al. reported that higher implant volumes were associated with greater reductions in NAC sensitivity, while Pitanguy et al. similarly observed more frequent sensory alterations in proportion to implant size.^17^^,^^42^ In fact, larger implants may increase tension across the breast envelope, generating subtle traction or compression on the fine sensory branches within the parenchyma and subareolar plexus.^17^ This relationship can be understood by analogy to naturally hypertrophic breasts, where excessive tissue weight and stretching lead to reduced cutaneous sensitivity.^66^ The degree of nerve strain likely depends less on absolute implant size and more on the proportion between implant volume and the available skin envelope, as larger devices placed within a tight envelope create greater tension and potential nerve injury than those in more compliant tissues.^66^ Thus, implant volume acts as a minor but plausible modifier of postoperative sensibility, primarily through biomechanical rather than neuroanatomical mechanisms.

Another notable methodological observation across the reviewed literature concerns the discordance between objective sensory testing and patient-perceived outcomes. Kompatscher and colleagues discussed this gap, highlighting that despite measurable elevations in pressure and vibration thresholds across all breast regions two years after transareolar augmentation, the majority of patients subjectively reported normal sensation.^50^ This can be explained by the unique anatomy of the NAC. Unlike glabrous skin, the nipple contains few classic mechanoreceptors, as Merkel cells are sparse and Meissner corpuscles are absent, while its sensitivity depends mainly on deeper smooth-muscle innervation and mechano-transduction within the glandular tissue.^19^ As a result, minor changes detected by monofilament or vibration testing may not be noticeable to patients, accounting for the frequent discrepancy between objective thresholds and subjective perception of nipple sensation. Similar methodological insights were demonstrated in reduction, where standardized Semmes-Weinstein testing showed predictable improvement in NAC sensation between 6 and 24 months, further supporting the relevance of objective tools for monitoring sensory recovery over time.^67^

An additional concept of interest is sensory neurotization, which refers to the identification and repair or reattachment of sensory nerve branches to support postoperative sensory recovery.^68^ This technique is explored primarily in post-mastectomy breast reconstruction, where extensive native nerve disruption may necessitate proactive sensory reinnervation.^68^, ^69^, ^70^ In this case, nerve coaptation between preserved intercostal sensory branches and the transferred tissue or NAC may be performed to restore afferent pathways.^71^^,^^72^ The procedure involves microsurgical nerve repair using epineural sutures or nerve grafts to reconnect the lateral intercostal nerve branches with the reconstructed breast envelope, thereby facilitating axonal regeneration and progressive sensory recovery.^71^^,^^72^ In contrast, primary cosmetic breast augmentation generally preserves most native neural pathways, and therefore neurotization techniques are underexplored and were not reported in the studies included in this review. Nonetheless, the concept remains relevant as sensory function becomes an increasingly recognized patient-centered outcome in breast surgery. Consistent with this broader emphasis on sensory preservation, newer tissue-preserving augmentation strategies, although not involving formal neurotization, have reported favorable early sensory outcomes, but require further comparative validation.^73^^,^^74^

### Strengths and limitations

This review provides a focused and comprehensive synthesis of postoperative sensibility involving both the NAC and regional breast skin after primary cosmetic augmentation, across surgical techniques. Methodological strengths include PRISMA 2020 adherence, an a priori eligibility framework restricted to primary augmentation in humans, dual independent screening and data extraction, and explicit capture of sensory methods (objective instruments vs patient-reported outcomes), surgical variables (incision, plane, implant characteristics), and early/long-term follow-up. By limiting to contemporary techniques (2000 - 2026) and organizing findings by clinically actionable domains, the review supports counseling and operative planning while identifying where standardized testing could strengthen future evidence. To preserve clinical and methodological comparability, the review excluded adjunctive or alternative procedures that introduce additional operative variables potentially affecting breast and NAC sensation, such as fat grafting, mesh/acellular dermal matrix use, or concomitant mastopexy. Comparative evaluation of sensory outcomes across these broader augmentation strategies remains an important area for future research. Future work should standardize endpoints for both NAC and quadrant-specific breast-skin sensibility (a core outcome set integrating validated objective measures with patient-reported outcome measures, such as *BREAST-Q* sensory items),^75^ require preoperative baselines and serial postoperative mappings, and stratify by incision micro-location, plane, and implant volume-to-envelope ratios. Multicenter prospective cohorts or randomized comparisons with longer follow-up are needed to clarify durability and define true risk modifiers.

The main limitations of this review stem from the predominantly observational nature of included studies, introducing potential selection and reporting biases. Heterogeneity in sensory assessment tools, timing, and test locations, combined with inconsistent definitions of incision and implant plane, limits comparability and prevents quantitative pooling. These limitations are further compounded by substantial methodological heterogeneity. Sensory outcomes were assessed using markedly different approaches, ranging from Semmes-Weinstein monofilaments, pressure-specified sensory devices, biothesiometry, and electrical threshold testing to clinical examination and patient-reported symptoms, with variable definitions of sensory alteration and recovery. The postoperative assessment ranged from the early weeks after surgery to several years of follow-up. Surgical reporting was similarly variable, with differences in the precise configuration and location of incisions and in the level of detail used to characterize implant planes, while implant volume, material, and surface characteristics were not uniformly reported across studies. Together, these differences limit direct cross-study comparison and preclude meaningful quantitative pooling. Moreover, key variables such as implant volume-to-envelope ratio and capsular changes were often underreported, as they were outside the main scope of this review, while discordance between objective and subjective measures complicates the interpretation. Importantly, the disproportionate numerical influence of two large cohorts that used non-standardized, narrative-based sensory assessment, could underestimate subtle or moderate sensory alterations, particularly when compared with objective modalities such as Semmes-Weinstein monofilaments or pressure-specified sensory devices. However, restricting interpretation to studies with defined sensory assessment methods and higher Newcastle-Ottawa Scale scores preserved the direction of the associations, supporting the stability of our findings independent of large narrative-based cohorts. An additional limitation is the absence of prospective protocol registration (e.g., with PROSPERO).

## Conclusion

This systematic review suggests that sensory alterations following primary breast augmentation are generally mild and transient, with near-complete recovery of nipple-areola and breast-skin sensation within the first postoperative year. The findings suggest that incision placement and implant plane may play a greater role in influencing postoperative sensibility than implant volume, with available evidence suggesting a relative advantage of inframammary and submuscular approaches for sensory preservation.

As the evidence base expands, future research should aim to standardize sensory assessment protocols and integrate validated patient-reported outcome measures. A deeper understanding of these factors is essential for guiding surgical planning, improving patient counseling, and optimizing both aesthetic and functional outcomes in breast augmentation surgery.

## Ethical approval

Not required.

## Funding

No funding was received for this article.

## Declaration of competing interest

Pietro G. di Summa is Deputy Editor of the Journal of Plastic, Reconstructive & Aesthetic Surgery. He had no involvement in the editorial handling, peer review, or decision-making process for this manuscript.
