# Supplementary material for: Breast augmentation and sensory outcomes: A systematic review of surgical techniques and their impact on nipple-areola complex and breast-skin sensibility
Source: JPRAS Open. 2026 Sep 29;52:626–46. doi: 10.1016/j.jpra.2026.09.028 (PMC13682357; doi:10.1016/j.jpra.2026.09.028)
Supplement: Supplementary file 1 [file mmc1.docx]

Supplementary Material

**Electronic Search Strategies**

*Breast Augmentation and Sensory Outcomes: A Systematic Review of Surgical Techniques and Their Impact on Nipple-Areola Complex and Breast-Skin Sensibility*

# Search methods and screening

A comprehensive electronic search was conducted in PubMed/MEDLINE, Scopus, Web of Science Core Collection, and Embase. The final search was run on **September 3, 2026**. Searches were restricted to English-language records and to publications from 2000 onward, using the database-specific date/year syntax shown below. Title/abstract screening and full-text eligibility assessment were performed independently by two reviewers using Rayyan. The database-specific Boolean strategies are reproduced below in full. Line breaks are included for readability and do not alter the Boolean logic.

**Search yield:** 1,622 records were identified before deduplication: PubMed/MEDLINE (n = 533), Scopus (n = 517), Web of Science Core Collection (n = 325), and Embase (n = 247). After merging the results, 737 duplicate records were removed, leaving 885 unique records for screening.

# Database search characteristics

| **Database / interface** | **Date searched** | **Date limits** | **Language limit** | **Records retrieved** |
| --- | --- | --- | --- | --- |
| PubMed/MEDLINE (PubMed) | 3 Sep 2026 | 1 Jan 2000–3 Sep 2026 | English | 533 |
| Scopus | 3 Sep 2026 | Publication years 2000–2026 | English | 517 |
| Web of Science Core Collection | 3 Sep 2026 | 1 Jan 2000–3 Sep 2026 | English (filter) | 325 |
| Embase | 3 Sep 2026 | Publication years 2000–2026 | English | 247 |

# Complete electronic search strategies

## 1. PubMed/MEDLINE

**Date searched:** 3 September 2026. **Records retrieved:** n = 533. **Limits:** Limits encoded in the strategy: publication date 1 January 2000 through 3 September 2026; English language.

(
 "breast augmentation"[tiab]
 OR "breast enlargement"[tiab]
 OR "augmentation mammoplasty"[tiab]
 OR "augmentation mammaplasty"[tiab]
)
AND
(
 "breast sensation"[tiab]
 OR "breast sensibility"[tiab]
 OR "breast sensitivity"[tiab]
 OR "nipple sensation"[tiab]
 OR "nipple sensibility"[tiab]
 OR "nipple sensitivity"[tiab]
 OR "nipple-areola complex"[tiab]
 OR "nipple-areolar complex"[tiab]
 OR
 (
 (breast[tiab] OR nipple*[tiab] OR areola*[tiab])
 AND
 (
 sensat*[tiab]
 OR sensory[tiab]
 OR sensib*[tiab]
 OR sensitiv*[tiab]
 OR numb*[tiab]
 OR hypoesthe*[tiab]
 OR hypaesthe*[tiab]
 OR paresthe*[tiab]
 OR paraesthe*[tiab]
 OR "perception threshold"[tiab]
 OR "perception thresholds"[tiab]
 )
 )
)
AND
("2000/01/01"[dp] : "2026/09/03"[dp])
AND english[la]

## 2. Scopus

**Date searched:** 3 September 2026. **Records retrieved:** n = 517. **Limits:** Limits encoded in the strategy: publication years 2000–2026; English language. The search was last run on 3 September 2026.

TITLE-ABS-KEY(
 "breast augmentation"
 OR "breast enlargement"
 OR "augmentation mammoplasty"
 OR "augmentation mammaplasty"
)
AND
TITLE-ABS-KEY(
 "breast sensation"
 OR "breast sensibility"
 OR "breast sensitivity"
 OR "nipple sensation"
 OR "nipple sensibility"
 OR "nipple sensitivity"
 OR "nipple-areola complex"
 OR "nipple-areolar complex"
 OR
 (
 (breast OR nipple* OR areola*)
 W/5
 (
 sensat*
 OR sensory
 OR sensib*
 OR sensitiv*
 OR numb*
 OR hypoesthe*
 OR hypaesthe*
 OR paresthe*
 OR paraesthe*
 OR "perception threshold"
 )
 )
)
AND PUBYEAR AFT 1999
AND PUBYEAR BEF 2027
AND LANGUAGE(english)

## 3. Web of Science Core Collection

**Date searched:** 3 September 2026. **Records retrieved:** n = 325. **Limits:** Date of publication restricted to 1 January 2000 through 3 September 2026. The English-language filter was applied in the Web of Science interface.

TS=(
 (
 "breast augmentation"
 OR "breast enlargement"
 OR "augmentation mammoplasty"
 OR "augmentation mammaplasty"
 )
 AND
 (
 "breast sensation"
 OR "breast sensibility"
 OR "breast sensitivity"
 OR "nipple sensation"
 OR "nipple sensibility"
 OR "nipple sensitivity"
 OR "nipple-areola complex"
 OR "nipple-areolar complex"
 OR
 (
 (breast OR nipple* OR areola*)
 NEAR/5
 (
 sensat*
 OR sensory
 OR sensib*
 OR sensitiv*
 OR numb*
 OR hypoesthe*
 OR hypaesthe*
 OR paresthe*
 OR paraesthe*
 OR "perception threshold"
 )
 )
 )
)
AND DOP=2000-01-01/2026-09-03

## 4. Embase

**Date searched:** 3 September 2026. **Records retrieved:** n = 247. **Limits:** Limits encoded in the strategy: publication years 2000–2026; English language. The search was last run on 3 September 2026.

(
 "breast augmentation":ti,ab,kw
 OR "breast enlargement":ti,ab,kw
 OR "augmentation mammoplasty":ti,ab,kw
 OR "augmentation mammaplasty":ti,ab,kw
)
AND
(
 "breast sensation":ti,ab,kw
 OR "breast sensibility":ti,ab,kw
 OR "breast sensitivity":ti,ab,kw
 OR "nipple sensation":ti,ab,kw
 OR "nipple sensibility":ti,ab,kw
 OR "nipple sensitivity":ti,ab,kw
 OR "nipple-areola complex":ti,ab,kw
 OR "nipple-areolar complex":ti,ab,kw
 OR
 (
 (
 (breast OR nipple* OR areola*)
 NEAR/5
 (
 sensat*
 OR sensory
 OR sensib*
 OR sensitiv*
 OR numb*
 OR hypoesthe*
 OR hypaesthe*
 OR paresthe*
 OR paraesthe*
 OR "perception threshold"
 )
 ):ti,ab,kw
 )
)
AND [2000-2026]/py
AND english:la

# Screening process

**Software:** Rayyan was used for title/abstract screening and full-text eligibility assessment. Two reviewers screened records independently, with disagreements resolved by discussion and consensus; a third reviewer was available to arbitrate unresolved disagreements.
