# Supplementary material for: Breast augmentation and sensory outcomes: A systematic review of surgical techniques and their impact on nipple-areola complex and breast-skin sensibility
Source: JPRAS Open. 2026 Sep 29;52:626–46. doi: 10.1016/j.jpra.2026.09.028 (PMC13682357; doi:10.1016/j.jpra.2026.09.028)
Supplement: Supplementary file 2 [file mmc2.docx]

*Supplementary Table S1: Study Quality Assessment Using the Newcastle-Ottawa Scale*

| Author and Year | Design | Selection (0-4) | Comparability (0-2) | Outcome (0-3) | Total (0-9) | Note |
| --- | --- | --- | --- | --- | --- | --- |
| Rancati et al., 2023 | Prospective cohort | 4 | 1 | 3 | 8 | Objective monofilament testing; direct incision comparison |
| Wieslander, 2020 | Retrospective cohort | 4 | 0 | 1 | 5 | Large cohort; narrative sensory assessment only |
| Ahmad, 2017 | Retrospective case series | 3 | 1 | 1 | 5 | Mixed approaches; subjective reporting |
| Lund et al., 2016 | Prospective multicenter cohort | 4 | 1 | 1 | 6 | Very large cohort; sensory outcomes captured via case report forms |
| Brown et al., 2016 | Prospective cohort | 4 | 0 | 3 | 7 | Objective monofilament testing; single incision type |
| Zhu et al., 2015 | Retrospective | 3 | 0 | 1 | 4 | Small cohort; subjective assessment |
| Mohmand and Ahmad, 2013 | Prospective observational | 3 | 0 | 1 | 4 | Subjective follow-up only |
| von Sperling et al., 2011 | Retrospective | 3 | 0 | 1 | 4 | Long-term outcomes; questionnaire-based |
| Araco et al., 2011 | Retrospective comparative | 4 | 2 | 2 | 8 | Large comparison; consistent clinical assessment |
| Niechajev et al., 2007 | Prospective | 4 | 1 | 2 | 7 | Structured tactile testing; multiple approaches |
| Pitanguy et al., 2007 | Prospective clinical | 3 | 1 | 3 | 7 | Combined objective and subjective testing |
| Sevin et al., 2006 | Retrospective case series | 3 | 0 | 1 | 4 | Large cohort; subjective reporting |
| Okwueze et al., 2006 | Prospective clinical trial | 4 | 1 | 3 | 8 | Incision comparison |
| Mofid et al., 2006 | Retrospective comparative | 3 | 1 | 3 | 7 | Objective testing; small sample |
| Stoff-Khalili et al., 2004 | Retrospective comparative | 3 | 1 | 1 | 5 | Subjective outcomes; plane comparison |
| Kompatscher et al., 2004 | Retrospective | 3 | 0 | 3 | 6 | Extensive objective testing; small cohort |
| Banbury et al., 2004 | Prospective | 4 | 0 | 3 | 7 | Objective and subjective testing; incision not reported |
| Ashraf et al., 2021 | Retrospective comparative | 3 | 1 | 3 | 7 | Random allocation to comparator planes; objective monofilament testing at 3 timepoints |
| Üstün et al., 2025 | Retrospective comparative with control group | 3 | 2 | 2 | 7 | Unoperated control group; objective 9-point monofilament protocol; biostatistician-adjusted analysis |
| Shi et al., 2015 | Retrospective comparative | 2 | 1 | 2 | 5 | Comparator group present but baseline age, BMI, and parity differed significantly between groups |
| Randquist et al., 2018 | Retrospective chart review | 2 | 0 | 2 | 4 | Small sample; patient-reported complaints rather than structured sensory testing; no comparator |
| Prado et al., 2008 | Prospective cohort | 2 | 1 | 2 | 5 | Objective faradic threshold testing; small sample; non-standard instrument |
| Kaasa et al., 2010 | Prospective RCT-derived cohort | 3 | 1 | 2 | 6 | Long (4-year) systematic follow-up of an RCT-derived cohort; prior perioperative pharmacological intervention |
| Balaguera Herrera et al., 2026 | Prospective cohort (novel technique) | 2 | 0 | 2 | 4 | Single-arm first-report series of a proprietary technique; no comparator group; sensation not tested with a validated instrument |
| Chacon-Quiros et al., 2026 | Prospective cohort (novel technique) | 2 | 0 | 2 | 4 | Single-arm, industry-sponsored first-report series of a proprietary device; sensory loss ascertained by passive complaint surveillance only |
| Stokes, 2004 | Retrospective questionnaire study | 2 | 0 | 1 | 3 | Selected cohort of thin patients; 68% questionnaire response; subjective sensory assessment; no comparator group. |
